# Trap-Assisted Charge Injection into Large Bandgap Polymer Semiconductors

**DOI:** 10.3390/ma12152427

**Published:** 2019-07-30

**Authors:** Dongdong Wang, Michael Fina, Suhan Kim, Chunmei Zhang, Ting Zhang, Yonghong Deng, Kai Chen, Lijuan Liang, Samuel S. Mao, Andrew M. Minor, Gao Liu

**Affiliations:** 1 Beijing Institute of Graphic Communication, Beijing 102600, China; 2 Energy Storage and Distributed Resources Division, Lawrence Berkeley National Laboratory, Berkeley, CA 94720, USA; 3 Department of Mechanical Engineering, University of California, Berkeley, CA 94720, USA; 4 National Center for Electron Microscopy, The Molecular Foundry, Lawrence Berkeley National Laboratory, Berkeley, CA 94720, USA; 5 School of Optoelectronics, Beijing Institute of Technology, Beijing 100081, China; 6 School of Chemistry and Chemical Engineering, South China University of Technology, Guangzhou 510640, China; 7 School of Materials Science and Engineering, Xi’an Jiaotong University, Xi’an 710049, China; 8 Department of Materials Science and Engineering, University of California, Berkeley, CA 94720, USA

**Keywords:** charge injection, trap, interface stability, polymer light-emitting diodes

## Abstract

The trap-assisted charge injection in polyfluorene-poly(3,4-ethylenedioxythiophene): poly(styrenesulfonate) (PEDOT:PSS) model systems with an Al or Al/LiF cathode is investigated. We find that inserting 1.3 nm LiF increases electron and hole injections simultaneously and the increase of holes is greater than electrons. The evolution of internal interfaces within polymer light-emitting diodes is observed by transmission electron microscopy, which reveals that the introduction of LiF improves the interface stability at both the cathode (cathode/polymer) and the anode (indium tin oxide (ITO)/PEDOT:PSS). Above-mentioned experimental results have been compared to the numerical simulations with a revised Davids model and potential physical mechanisms for the trap-assisted charge injection are discussed.

## 1. Introduction

Polymer light-emitting diodes (PLED) have attracted widespread attention ever since they were first reported by Burroughes et al. in 1990 [1], for their advantages such as solution-process method, low-cost, light weight, and flexibility. Knowledge of the physical processes governing the operation of PLED is essential for optimizing the device performance. Due to the low carrier concentration present in semiconducting polymers, crucial for efficient operation of PLEDs are the adequate and balanced charge injections of both electron and hole, which are controlled by the injection barriers from respective electrode contacts. In the Mott–Schottky limit, electron-injection barrier *Φ_B,e_* is defined by the difference between the cathode work function and the polymer lowest unoccupied molecular orbital (LUMO), and hole-injection barrier *Φ_B,h_* by the difference between the anode work function and the polymer highest occupied molecular orbital (HOMO). Depending on the magnitude of barrier height, the current can be either injection limited or space-charge limited, and a big interface barrier at either the electron or hole contact may hinder the injection process which results in excess carriers of one polarity and consequently decreasing the recombination efficiency significantly. In order to optimize the charge injection properties, two main approaches have been explored to minimize the injection barriers: decreasing cathode work function to improve electron injection, commonly achieved by using low-work-function metals such as Mg and Ca and their alloys or composite cathodes such as LiF/Al, and increasing anode work function to favor hole injection, which is often represented by introducing poly(3,4-ethylenedioxythiophene):poly(styrenesulfonate) (PEDOT:PSS) on top of indium tin oxide (ITO) anode [2].

Efficient and long-lasting blue light-emitting is necessary to obtain full-color displays and white solid-state lighting, since green and red light may be produced through doping or phosphors using photon down-conversion. However, because of the high contact barriers arising from the large bandgap (~3 eV) between the HOMO and LUMO levels of blue-light-emitting polymers, minimizing their injection barriers and balancing their charge injections poses greater challenge compared with red and green ones. For example, one of the most widely studied classes of blue-light-emitting polymers is polyfluorene (PFO). The mismatch between the work function of common electrodes (e.g., PEDOT:PSS and Al) and the molecular energy levels of PFO is huge, ranging from 0.6–1.7 eV [2,3,4]. Recently, a number of studies have reported the trap-assisted charge injections into large bandgap polymer semiconductors [4,5,6,7,8,9,10,11,12,13]. With the accumulation of electrons close to anode by filling the electron trap states (ETS), the PLEDs can be switched “on” and an ohmic hole contact is formed despite the large hole-injection barriers. Ohmic hole injection was reported by Woudenbergh et al. and Poplavskyy et al. [4,9]. Electric field screening effects and the role of injected electrons were discussed by Lane et al. and Brewer et al. using electromodulation spectroscopy to probe the internal electric field [5,6,7,8]. Murata et al. and Bange et al. found that double-carrier devices showed much larger currents than hole-only devices and proposed the presence of a barrier to electron extraction at anode [10,13]. PEDOT:PSS trap states were measured and determined to be electron traps by Kanaan et al. and Nguyen et al [11,12]. However, due to the absence of direct characterization techniques, a general understanding of the electron-enhanced hole injection, as well as physical models describing the injection process, is still lacking. In this work, we study the PFO-PEDOT:PSS model systems with an Al or Al/LiF cathode. The current density-voltage (*J-V*) characteristics and calculations based on Richardson-Schottky (RS) model show that 1.3 nm LiF layer makes the electron-injection barrier height decrease from 1.12 eV to 0.89 eV. Meanwhile, the electroluminescence (EL) current efficiency anomalously decreases, which indicating that the enhanced electron injection into a hole-dominant PLED gives rise to a worsened carrier balance. Since LiF interlayer improves the device stability, we employ a new developed shadow-focused ion beam (sFIB) [14,15,16] method to prepare cross-sectional specimens and use transmission electron microscopy (TEM) to evaluate the interface deterioration. TEM results surprisingly reveal that the 1.3 nm LiF layer improves the interface stability at both the cathode (cathode/polymer) and the anode (ITO/PEDOT:PSS). These results have been compared to the numerical simulations with a revised Davids model [17,18] and a physics mechanism for the electron-enhanced hole injection is proposed. Our results suggest a new promising way to realize efficient charge carrier injections into semiconducting polymers despite large barriers.

## 2. Materials and Methods

Organic materials we used here included a PFO-based blue light-emitting polymer, poly (9,9-di(2-(2-(2- methoxy-ethoxy)ethoxy)ethyl)fluorenyl-2,7-diyl) (America Dye Source Inc., Baie d’Urfé, Canada, with a *M_w_* of 64,000 Da and a polydispersity of 7.4), and PEDOT:PSS (Bayer, Leverkusen, Germany, AI-4083). The ITO-coated glasses with a sheet resistance of 20 Ω/square were cleaned sequentially in ultrasonic bath of acetone, ethanol and deionized water, and then blown dry with air. PEDOT:PSS was coated by spin casting and heated at 150 °C for 30 min (40 nm thick). A 15 mg/mL PFO solution in chlorobenzene was used for the coating of light-emitting layer (60 nm thick). LiF and Al cathode were deposited by thermal evaporation in a vacuum chamber under a pressure of ~8 × 10^−7^ Torr. The anode and cathode together define a device of 0.08 cm^2^ and four identical devices are made simultaneously on the ITO-coated glass.

The thickness of PEDOT:PSS and PFO layers were determined by averaging multiple measurements with a Dektak surface profiler. The thickness of LiF films was monitored by a quartz crystal oscillator. For electrical measurement, a Keithley Sourcemeter (model 2420, Cleveland, OH, USA) was used and the luminance characteristics were investigated with a Minolta luminance meter (model LS-110, Osaka, Japan). For the TEM specimen preparation, a FEI Strata 235 dual beam FIB (FEI, Hillsboro, OR, USA) was used. For the TEM analysis a 300KV JEOL 3010 TEM (JEOL, Tokyo, Japan) and Zeiss Libra 200 energy-filter TEM instruments (Oberkochen, Germany) were employed.

## 3. Results and Discussion

**Figure 1:**
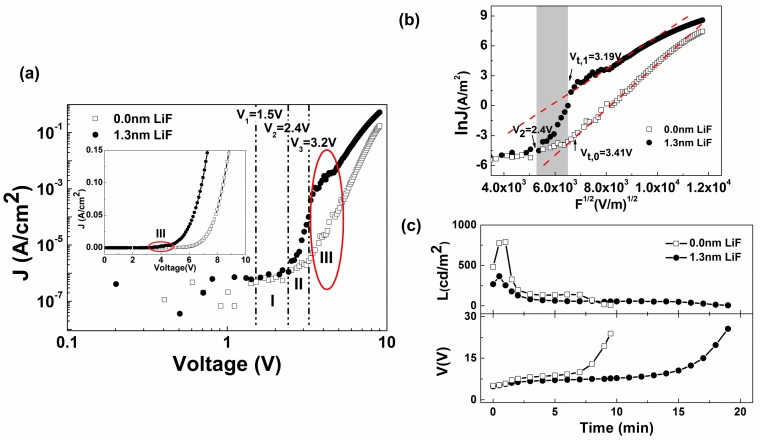
(**a**) *J-V* characteristics of the pristine and LiF polymer light-emitting diodes (PLEDs) on a log-log scale. The inset shows the curves on a linear scale. The three dashed lines represent the expected voltages at which holes begin to inject (*V_1_* = *V_bi_* + *Φ_B,h_*), electrons begin to inject (*V_2_* = *V_bi_* + *Φ_B,e_*), and light can be given out (*V_3_* = *E_g_*/*e*) for the pristine device. A built-in potential of 0.7 eV was taken into consideration. The circled region (Region III) possesses the turn-on voltage range of the two PLEDs. (**b**) Richardson–Schottky plot for the *J-V* characteristics centered by Region II (grey region) of Figure 1. (**c**) Luminance decay and voltage rise properties of the pristine and LiF devices at a constant-current-density operation of 40 mA/cm^2^.

Figure 1a shows the *J-V* characteristics of the two PLED devices: ITO/PEDOT:PSS/PFO/Al and ITO/PEDO T:PSS/PFO/LiF(1.3 nm)/Al. The PLED without LiF is designated as the pristine device and the other one as LiF device. As shown in the inset, inserting a LiF film enhanced the current density significantly. On a log-log scale the *J-V* curves of the pristine PLED can be divided into three distinct regions (see Figure S1) [19,20,21].

(1) $$V>V_{bi}+\Phi_{B,h}:h^{+}$$

(2) $$V>V_{bi}+\Phi_{B,e}:h^{+}+e^{-}$$

(3) $$V>E_{g}/e:\;h^{+}+e^{-}\to hv$$

where *V* is the applied bias, *V_bi_* the built-in voltage, *Φ_B,h_* the hole barrier, *Φ_B,e_* the electron barrier, and *E_g_* the bandgap of PFO. The smaller of the two barriers controls the initial *J-V* characteristics, and the larger one controls the *J-V* characteristics in the EL condition [22,23]. In region I, holes are injected into PFO layer first. The *J-V* curves of the two PLEDs are almost coincident since *Φ_B,h_* doesn’t change. In region II, electron injection occurs and the current through the LiF device begin to increase much sharper than that through the pristine device. This implies that the influence of LiF interlayer on electron injection is the origin for the difference in *J-V* profiles. It is worth noting that in region II, the injected holes and electrons are not able to form excitons to produce light emission since the applied voltage here is less than the energy bandgap of PFO. In region III, appreciable numbers of electrons are injected and meet dominant holes in PFO to form excitons and measurable EL is perceived. The positions denoted by arrows, *V_t,_*_0_ and *V_t,_*_1_, correspond to the different turn-on voltages (Figure 1b), which are defined by the voltage at which a measurable luminescence (1 cd/m^2^) is detected. *V_t,_*_0_ is 3.41 V for the pristine device, and *V_t,_*_1_ is 3.19 V for the LiF device.

Where injection barrier heights are larger than ~0.3–0.4 eV, the current flow is injection limited [17]. The *J-V* characteristics shown in the grey Region of Figure 1b bear out a power-law behavior of *j∝V^3^* for the pristine device and *j∝V^21^* for the LiF device. While the large power–law relation may be regarded as indicative of trap-controlled space-charge-limited current (SCLC), this has to be considered accidental (see Figure S3) as the higher barriers exist in our devices. We describe the electron injection characteristics in terms of the RS model for thermionic emission [24,25], and the *J-V* characteristics are given by

(4) $$J=AT^{2}exp\left[-\frac{\Phi_{B,e}-\sqrt{e^{3}F/4\pi \varepsilon_{0}\varepsilon_{r}}}{kT}\right]$$

where *A* is the Richardson constant, *T* the temperature, *k* the Boltzmann constant and *F* the internal strength of electric field. Figure 1b shows the RS model plots for the *J-V* curves of *ln|J| vs F^1/2^*. By extrapolating the linear-fit lines (the two red dotted lines) to F = 0, the barrier height for electrons injection was obtained, which was 1.12 eV for the pristine device and decreased to 0.89 eV for the LiF device. In line with expectations, inserting a LiF interlayer decreased the electron injection barrier and turn-on voltage significantly. According to the general expectations, an improved balance of charge carriers and higher EL efficiency arising from the enhanced electrons injection to a hole-dominant PLED device should be reached. However, this is not the case.

Both the pristine and LiF devices were electrically aged continuously in a nitrogen-filled glove box at a constant current density of 40 mA/cm^2^ until no measurable light outputs were detected. The luminance decay and voltage rise properties accompanied by the degradation of the two devices are shown in Figure 1c. It can be seen that the LiF interlayer significantly improves the device stability as the luminance decay and voltage increase are both much slower than pristine one. On the other hand, it is *abnormal* that at the given current density LiF device exhibits less light emission. The lower current efficiency suggests a worse charge carrier balance despite the enhancement of electron injection into a hole-dominant device, which indicates that hole injections is enhanced simultaneously and the increase of holes is greater than electrons.

**Figure 2:**
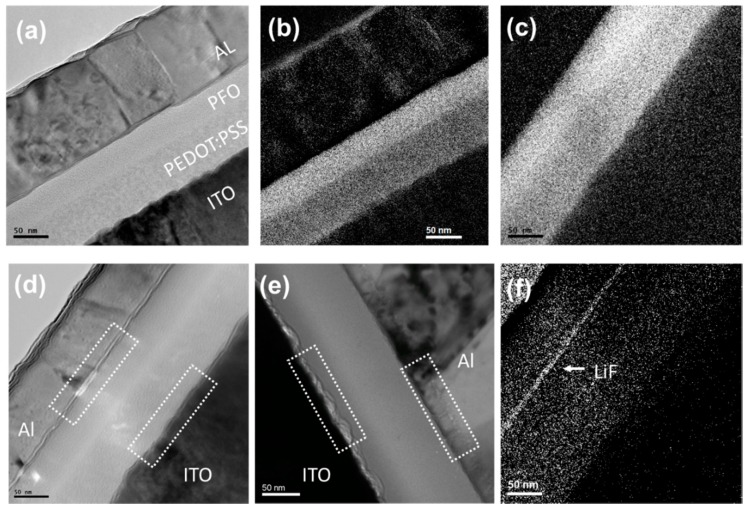
Cross-sectional TEM images for (**a**) fresh LiF device; (**b**) fresh LiF device under carbon map; (**c**) operated LiF device under carbon map; (**d**) operated LiF device; (**e**) operated pristine device (**f**) operated LiF device under Lithium map.

PLED device owns a multilayered structure and the interfaces between these layers play a crucial role for the injection, localization, and recombination of charge carriers. TEM possesses the unique ability to provide detailed chemical and structural information down to nanoscale [26,27]. However, the requirement on specimens for being transparent to electrons make it difficult to investigate a working PLED, which is composed of ITO-coated glass, polymer layers and metal electrode, varying from metallic, organic to inorganic in material and from nanometers to millimeters in thickness. Using a recently developed sFIB method (see Figure S2), we obtained high-quality cross-sectional PLED specimens with a thickness of about 100nm, which make us be able to evaluate the interface deterioration. A TEM image of the fresh LiF device is shown in Figure 2a. ITO, PEDOT:PSS, PFO and Al layers in the stack are clearly visible and easily localized. The images demonstrate no visible damage, such as shrinking or stretching, to the multilayer structure occurs during specimen preparation. Lithium elemental mapping from electron energy loss spectroscopy analysis using the three window method shows that the 1.3 nm LiF layer between Al and PFO is a continuous thin film (Figure 2f). Using carbon mapping, the fresh PEDOT:PSS/PFO interface (Figure 2b) appears sharp and smooth, which suggests no mixing happens between PEDOT:PSS and PFO during device fabrication. This is in line with expectations since each polymer is spun casting from solvent with very different polarity (water and chlorobenzene). The carbon map of operated PEDOT:PSS/PFO interface (Figure 2c) becomes blurry, which means a certain degree of polymer intermixing happened. Similar phenomena were observed for the PEDOT:PSS/PFO interface in pristine device. A dramatic difference in the degradation of ITO/ PEDOT:PSS and cathode/PFO interfaces is shown in Figure 2d,e. For the operated LiF device, there was no obvious damage that occurred at the ITO/PEDOT:PSS and Al/LiF/PFO interfaces until the device failure. For the operated pristine device, however, some morphological structures on nanoscale occurred along the ITO/PEDOT:PSS and Al/PFO interfaces. Surprisingly, inserting a LiF Layer between Al and PFO improves the interface stability of cathode/PFO and ITO/ PEDOT:PSS simultaneously.

**Figure 3:**
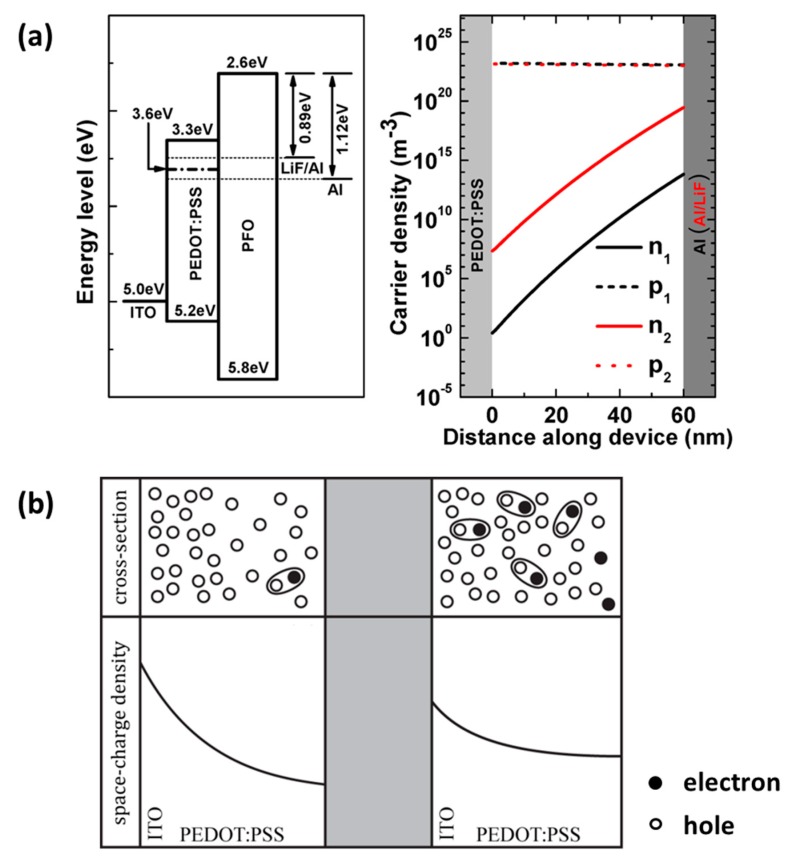
(**a**) Energy level diagram (left) and charge carrier profiles in polyfluorene (PFO) layer (right). (**b**) Schematic representations describing the proposed electron-enhanced hole injection mechanism. PEDOT:PSS layer in pristine device (left panel) and LiF device (right panel), respectively. A significant number of electrons begin to penetrate into the PEDOT:PSS, leading to lowered ITO/PEDOT:PSS interfacial space charges and raised bulk space charges in PEDOT:PSS compared to the pristine device.

To provide some physical insights into the decrease of EL efficiency and the improved interfacial stability of ITO/PEDOT:PSS, we investigated the energy diagram and the charge carrier profiles (Figure 3a). It’s interesting that the effective energy level of Al/LiF cathode lies above the ETS located in PEDOT:PSS, but that of Al cathode lies under the ETS. For the calculation of carrier density profiles, we consider the PLED a single-layer device of PFO and use the equivalent electrode work functions, ITO/PEDOT:PSS as anode and Al (or Al/LiF) as a cathode. Our model is based on the common Davids model [17], but the carrier recombination is treated as a hopping process [18]. The numerical results show that varying the electron injection barrier *Φ_B,e_* from 1.12 eV to 0.89 eV results in that the electron density in PFO layer increases ~10^6^ times. Although the LiF device remains hole-dominant, and the electron density is still negligible when compared with hole density (less than 0.1%), it is worth pointing out that in PFO layer close to PFO/PEDOT:PSS interface, there are far fewer electrons for the pristine device (~10^0^/m^3^) than for the LiF device (~10^7^/m^3^). The injected electrons from cathode seems totally confined inside the PFO layer for the pristine device, but are able to flow throughout the whole PFO layer for the LiF device.

The improvement of cathode/PFO interfacial stability with 1.3 nm LiF can be understood since the improved electron injection will move the recombination zone away from the cathode, which may be attributed to reduce hole accumulation near the cathode [19,28]. For the ITO/PEDO:PSS interface, we propose a physics mechanism here (Figure 3b) based on the experimental and numerical results discussed above. The ITO/PEDOT:PSS interface was able to produce quasi-SCLC hole injection [29]. Due to the highly efficient hole injection through ITO/PEDOT:PSS interface and the 0.6 eV barrier at PEDOT:PSS/PFO interface, the accumulation of holes inside PEDOT:PSS layer redistribute the electric field and are primarily displaced through hole diffusion [30]. Inserting a 1.3 nm LiF layer improves the electron injection, and electrons begin to flow throughout the whole PFO layer. The lack of an electron barrier at the PFO/PEDOT:PSS interface leaves electrons reaching the interface flow unhindered into PEDOT:PSS, and begin to fill the ETS. Two consequences arise from filling the ETS, (1) the electron mobility in the PEDOT:PSS layer begins to increase significantly as the electron quasi-Fermi level moves towards and through the ETS energy level [30]. Further, it is then the ability of additional electrons injected into PEDOT:PSS layer to, on average, increase the electron population in the neighborhood of ITO which modifies the space charge profile in the PEDOT:PSS layer (Figure 3b). (2) The net space charge is reduced as electrons and trapped electrons begin to compensate the hole density. Since ITO/ PEDOT:PSS interface is capable of SCLC hole injection, the number of holes injected through this interface will increase to supply the PEDOT:PSS layer. Physically, this means that more electrons shield holes, in turn, enabling larger hole injection current. Finally, this shielding effect allows holes to drift away from the ITO/PEDOT:PSS interface, which in turn leads to a more moderate surface to the interface charges.

Either introducing PEDOT:PSS on top of ITO [31] or inserting thin LiF film between cathode and organic layer [32] has become a common practice nowadays. Our results shed light on the complexity of the mechanisms and illustrate how much the mechanism depends upon the material systems and device architecture. For the PEDOT:PSS, its acidic nature etching the underlying ITO was long-thought to be responsible for device degradation. Consistent with the previous finding that neutralizing PEDOT:PSS with NaOH solution cannot lead a better lifetime and performance [33], our result suggests that the influence of local accumulation of immobile charges [19] is more important than the acidity issue for interface degradation. For the LiF, the thickness of LiF was increased progressively and the EL efficiency and lifetime were recorded as a function of the LiF thickness. Moreover, there appears to be a trade-off between EL current efficiency and lifetime. Again, all devices were run under a constant current density of 40 mA/cm^2^. The device with 2.7 nm-thick LiF layer showed the longest lifetime. However, the EL efficiency began to be much higher than the pristine one at 4.4 nm LiF, increased further until reaching a maximum value at 7.6 nm and then deteriorated with thicker LiF. These results were consistent with previous experimental results of PLED with similar device structure [34], and suggested that when inserting around 4.4 nm or thicker LiF, the LiF interfacial layer at cathode may play a hole-blocking role [35] besides enhancing the electron injection for the higher EL efficiency. Finally, our results gave a conclusive answer why the effect of trap-assisted injection for PFO is specifically observed for the PFO-PEDOT:PSS system but not for other anode materials [4], since the occupation status of ETS and redistribution of space charge profile located in PEDOT:PSS determined the enhance hole injection.

## 4. Conclusions

In summary, we have studied the PFO-PEDOT:PSS model systems with Al or Al/LiF cathode. The results show inserting 1.3 nm LiF increases electron and hole injections simultaneously. Moreover, TEM results reveal that the interfacial stability of both ITO/PEDOT:PSS and cathode/PFO improve meanwhile. The ETS located in PEDOT:PSS and their occupation status are regarded as playing a key role, and the physics mechanism is proposed. It is worth noting that although most investigations of electron-enhanced hole injection were focused on PFO-PEDOT:PSS model systems [4,5,6,7,8,9,10,11,12,13], this effect was observed in other polymers with other anode contacts [36]. More recently, the hole-enhanced electron injection from amorphous ZnO in inverted PLED was also reported [37]. The trap-assisted charge injection offers a promising way to obtain efficient charge carrier injection despite large barriers. For example, electron traps or hole traps may be introduced close to the counter electrode on purpose to improve bipolar charge carriers injection simultaneously, which is especially interesting for large bandgap blue PLEDs.

## Supplementary Materials

The following are available online at https://www.mdpi.com/1996-1944/12/15/2427/s1, Figure S1: Energy level diagram for the pristine PLED. The HOMO and LUMO levels of PEDOT:PSS and PFO are taken from the following references [1,2,3,4]; Figure S2: (a) Schematic of the sFIB technique for cross-sectional TEM sample preparation. In this case, a thin film (red) is tilted so that the surface is in the “shadow” of the ion beam. This geometry allows for the ability to cross-section a sample without using a sacrificial layer or long milling times. The ion beam is focused on either side of a corner of the sample, resulting in an electron-transparent wedge ending on the film surface. The angle of incidence of the ion beam can be varied depending on the circumstances, as long as it is directed from the back, substrate-side. (b) SEM figure of the cross-sectional PLED TEM specimen produced by the Shadow-FIB milling geometry; Figure S3: Experimental J-V characteristics of the pristine and LiF PLED with the theoretical SCLC curve on a linear scale. The J-V curves of both devices are not able to fit Mott-Gurney Law.
