# Supplementary material for: Trap-Assisted Charge Injection into Large Bandgap Polymer Semiconductors
Source: Materials (Basel). 2019 Jul 30;12(15):2427. doi: 10.3390/ma12152427 (PMC6695836; doi:10.3390/ma12152427)
Supplement: Supplementary file 1 [file materials-12-02427-s001.pdf]

materials

Supplementary Materials: Trap-Assisted Charge Injection into Large Bandgap Polymer Semiconductors

Dongdong Wang [1,2], Michael Fina [3], Suhan Kim [4], Chunmei Zhang [1,2], Ting Zhang [2,5],, Yonghong Deng [2,6], Kai Chen [2,7], Lijuan Liang [1], Samuel S. Mao [3], Andrew M. Minor [4,8,*] and Gao Liu [2,*]

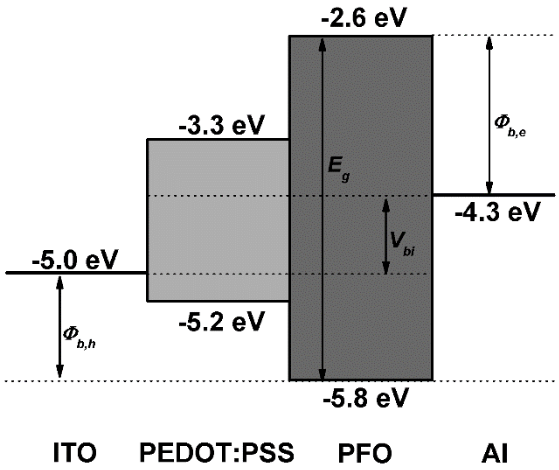

Figure S1. Energy level diagram for the pristine PLED. The HOMO and LUMO levels of PEDOT:PSS and PFO are taken from the following references [1–4].

In the absence of Fermi level pinning and space charges, the electric field inside PLED is given by $(V-V_{bi})/D$, where V is the applied bias, D the thickness of organic semiconductor layer and V_{bi} the built-in voltage. V_{bi} may be expressed as $V_{bi} = (W_a - W_c)/e$, where e is electron charge, W_a the anode work function and W_c the cathode work function. V_{bi} was taken as 0.7 V from the difference between work functions of ITO (5.0 eV) and Al (4.3 eV). Under a small applied bias V ($V < V_{bi}$), the internal electric field is negative (directed from cathode to anode) and the PLED behaves as a capacitor. When the applied voltage reached V_{bi} ($V = V_{bi}$), the internal field and stored charges vanished. If the applied voltage increased further ($V > V_{bi}$), the internal electric field became positive and carriers began injection. It is well known that the electron and hole injections are respectively controlled by the injection barriers $\Phi_{B,e}$ and $\Phi_{B,h}$. In the Mott-Schottky limit, $\Phi_{B,e}$ is defined by the difference between the cathode work function and the PFO LUMO (2.6 eV), and $\Phi_{B,h}$ by the difference between the ITO work function and the PFO HOMO (5.8 eV). The smaller of the two barriers controls the initial J-V characteristics, and the larger one controls the J-V characteristics in the EL condition. In our case, the larger barrier, $\Phi_{B,e}$, is ~1.7 eV at the Al (4.3 eV)/PFO interface (The value may be a bit smaller for Al/LiF/PFO contact). For the ITO (5.0 eV) /PEDOT:PSS (5.2 eV)/PFO contact, $\Phi_{B,h}$ is ~0.8 eV. As a result, the expected bias at which injection starts may be expressed as $V_1 = V_{bi} + \Phi_{B,h}$ for holes and $V_2 = V_{bi} + \Phi_{B,e}$ for electrons. Moreover, the expected turn-on voltage may be expressed as $V_3 = E_g/e$ where E_g is the bandgap between the LUMO and HOMO levels of PFO. V_1, V_2 and V_3 are exhibited as the dashed lines in Figure 1.

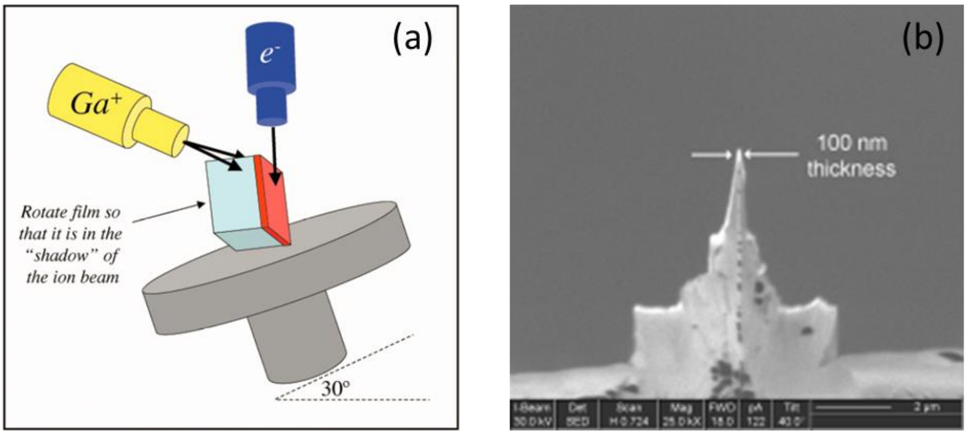

Figure S2. (**a**) Schematic of the sFIB technique for cross-sectional TEM sample preparation. In this case, a thin film (red) is tilted so that the surface is in the "shadow" of the ion beam. This geometry allows for the ability to cross-section a sample without using a sacrificial layer or long milling times. The ion beam is focused on either side of a corner of the sample, resulting in an electron-transparent wedge ending on the film surface. The angle of incidence of the ion beam can be varied depending on the circumstances, as long as it is directed from the back, substrate-side. (**b**) SEM figure of the cross-sectional PLED TEM specimen produced by the Shadow-FIB milling geometry.

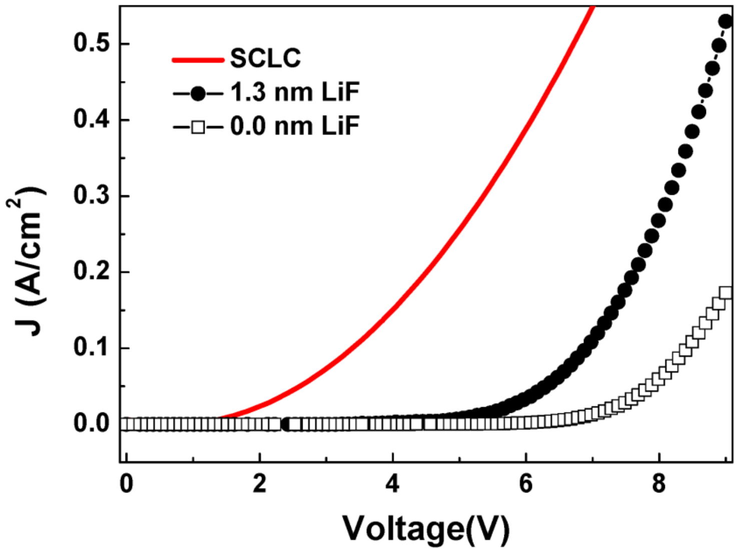

Figure S3. Experimental J-V characteristics of the pristine and LiF PLED with the theoretical SCLC curve on a linear scale. The J-V curves of both devices are not able to fit Mott-Gurney Law.

References

1. Son, D.; Kwon, B.; Park, D.; Seo, W.; Yi, Y.; Angadi, B.; Lee, C. and Choi, W. Emissive ZnO-graphene quantum dots for while-light-emitting diodes. *Nat. Nanotechnol.* **2012**, *7*, 465–471.
2. Woudenbergh, T.V.; Wildeman, J.; Blom, P.W.M.; Bastiaansen, J.J.A.M.; Langeveld-Voss, B.M.W. Electron-enhanced hole injection in blue polyfluorene-based polymer light-emitting diodes. *Adv. Funct. Mater.* **2004**, *7*, 677–683.
3. Perumal, A.; Faber, H.; Yaacobi-Gross, N.; Pattanasattayavong, P.; Burgess, C.; Jha, S.; McLachlan, M.; Stavrinou, P.; Anthopoulos, T.; Bradley, D.D.C. High-Efficiency, Solution-Processed, Multilayer Phosphorescent Organic Light-Emitting Diodes with a Copper Thiocyanate Hole-Injection/Hole-Transport Layer. *Adv. Mater.* **2015**, *27*, 100.

4. Neher, D. Polyfluorene Homopolymers: Conjugated Liquid-Crystalline Polymers for Bright Blue Emission and Polarized Electroluminescence. *Macromol. Rapid Commun.* **2001**, *22*, 1365–1385.
